# Supplementary material for: Immature Sword Bean (Canavalia gladiata) Pod Alleviates Allergic Rhinitis (A Double-Blind Trial) Through PI3K/Akt/mTOR Signaling
Source: Nutrients. 2025 Jan 28;17(3):468. doi: 10.3390/nu17030468 (PMC11820081; doi:10.3390/nu17030468)
Supplement: Supplementary file 1 [file nutrients-17-00468-s001.zip › nutrients-3427814-supplementary.pdf]

Supplementary Figure S1: Effects of SBP extracts on the protein expression of PI3K/Akt/mTOR-signaling molecules in EOL-1 cells.

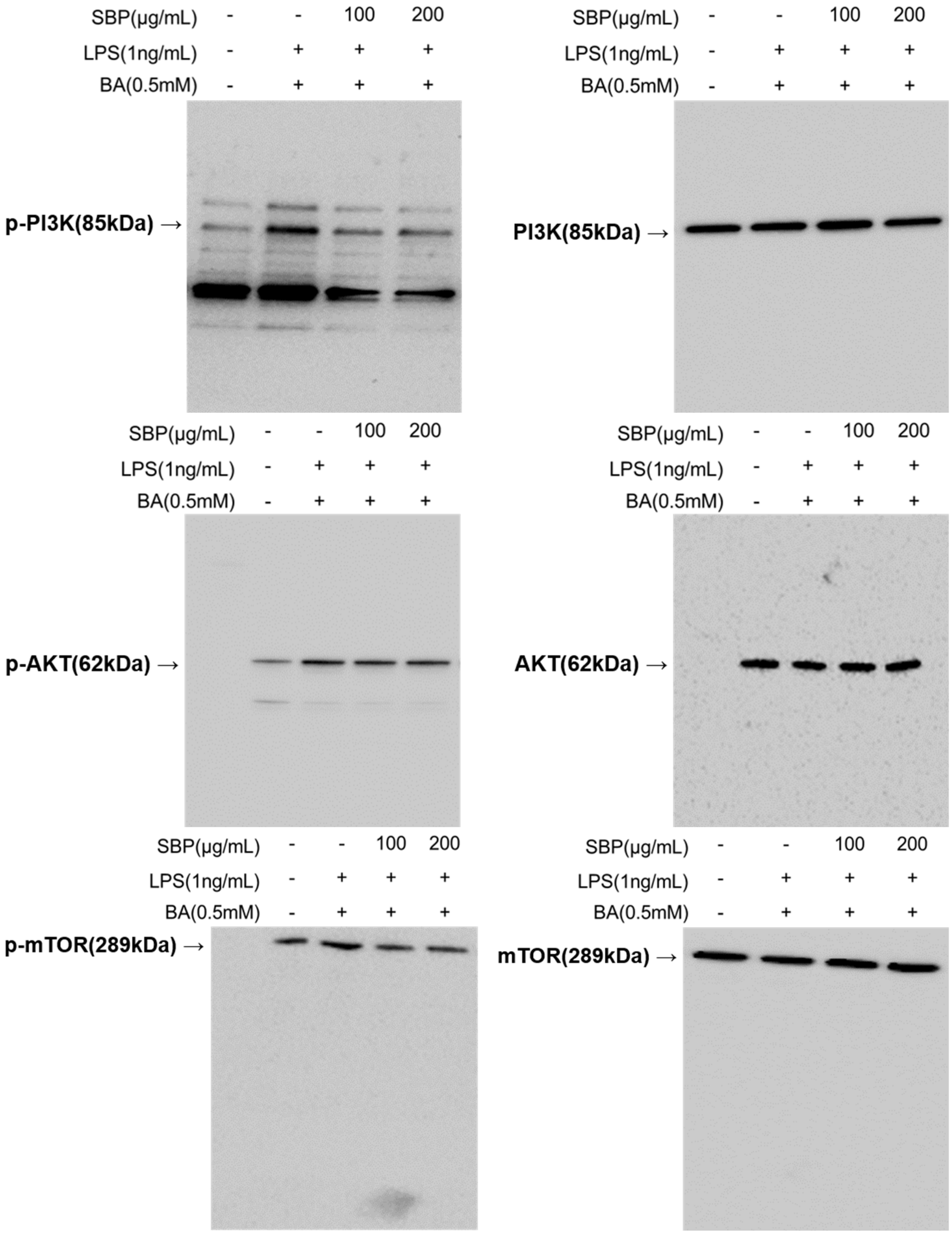

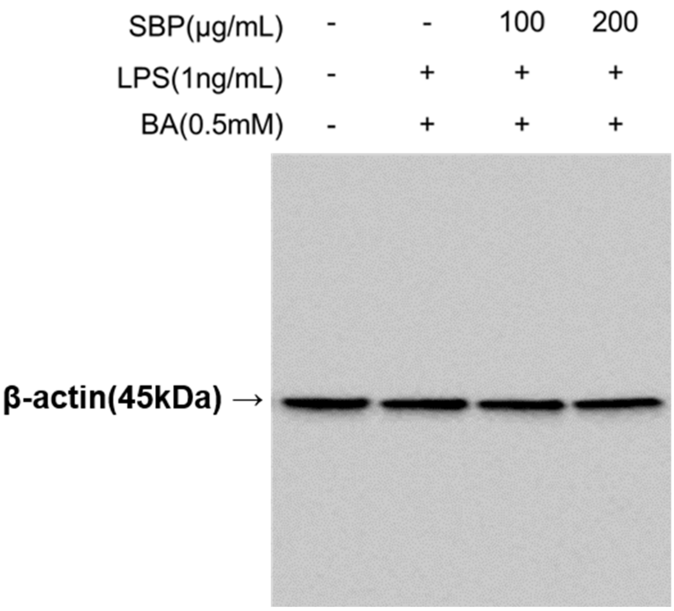
SBP(μg/mL) - - 100 200
LPS(1ng/mL) - + + +
BA(0.5mM) - + + +
β-actin(45kDa) →
